# The variation in shape and thickness of the pelvic floor musculature in males and females: a geometric-morphometric analysis

**DOI:** 10.1007/s00192-022-05311-5

**Published:** 2022-08-05

**Authors:** Ekaterina Stansfield, Philipp Mitteroecker, Wolfgang Umek, Barbara Fischer

**Affiliations:** 1 Department of Evolutionary Biology, University of Vienna, Djerassiplatz 1, 1030 Vienna, Austria; 2 Department of Obstetrics and Gynaecology, Medical University of Vienna & Karl Landsteiner Institute for Special Gynaecology and Obstetrics, Vienna, Austria

**Keywords:** Pelvic floor muscle shape, Geometric morphometrics, Pelvic floor disorders

## Abstract

**Introduction and hypothesis:**

In women, the risk of pelvic floor prolapse is known to be associated with age and parity. Different studies suggested that it is also related to pelvic dimensions, e.g. biomechanical modelling showed that a larger pelvic canal results in higher values of displacement, stress and strain in the pelvic floor muscles, which can increase the risk of pelvic floor disorders. To better understand the multiple factors contributing to pelvic floor disorders, we assessed how age, body weight, body height, parity (in women), pelvic canal size and overall muscle development affected pelvic floor geometry.

**Methods:**

A comprehensive geometric morphometric analysis of variation in pelvic floor muscle shape was conducted based on a dense set of 3D landmarks measured on CT scans in a cohort of 49 deceased men and 52 deceased women. The multivariate association between biological variables (parity, dimensions of the true pelvis, age, body weight, height) and pelvic floor muscle morphology was explored by reduced rank regression in both sexes.

**Results:**

In women, advanced age, high body weight relative to body height and a large pelvic canal were associated with a deeper pelvic floor. Surprisingly, parity did not have any strong association with overall pelvic floor shape. In men, high body weight was associated with a deep pelvic floor. Age had little effect on male pelvic floor shape, except for the thickness of the ischiocavernosus muscle, which reduced with age.

**Conclusion:**

These results suggest that age, relative body weight and the size of the pelvic canal contribute to the risk of female pelvic floor disorders via their effect on pelvic floor shape, independently of birth-related factors such as injury and avulsion of pelvic floor muscles.

**Supplementary information:**

The online version contains supplementary material available at 10.1007/s00192-022-05311-5.

## Introduction

Pelvic floor disorders (PFDs) are a common health issue for women, especially as they age. The pooled prevalence of PFDs has been estimated to range from 22 to 29% for women [1] but can be as high as 68% according to some reports [2]. PFD is a summary term for different disorders, including urinary incontinence, faecal incontinence and pelvic organ prolapse (physical descent of the pelvic organs through the vagina or rectum). PFDs occur when the muscle diaphragm of the pelvic floor and its connective tissue, which support the pelvic organs, are weakened or damaged. Epidemiological studies have shown that parity (number of childbirths) and birth mode, especially vaginal birth, are important risk factors for women to develop PFD [2, 3]. Ageing processes and trauma, which result in reduced elasticity and anatomical alteration of the pelvic floor muscles, can also contribute to the risk of PFD, independent of parity [4].

In men, the prevalence of PFDs is generally much lower. Surgery and trauma are among the most frequent risk factors for PFD [5]. Age, genetic background, increased intra-abdominal pressure, obesity and a high body mass index (BMI) are, however, among the PFD risk factors shared by both sexes [6].

There exists some evidence in the literature that women with a broader pelvic canal are more prone to developing incontinence and prolapse [7], but other studies failed to find such associations [8]. In a finite element analysis, Stansfield et al. [9] modelled the female pelvic floor muscles and demonstrated that a larger pelvic canal results in higher values of displacement, stress and strain in the pelvic floor muscles, which could contribute to a higher risk of PFD. In the same study, they showed that this effect is partially ameliorated by increased pelvic floor muscle thickness.

Although the epidemiological literature reports PFD prevalence and risk factors from large population surveys [2, 3], most clinical studies concentrate on the typical male and female anatomy of the pelvic floor and on the pathological features in persons with PFD. A number of clinical studies reported on morphological variation in the pelvic floor that is relevant for the development of PFD [10, 11]. In the present study, we aim to extend current knowledge about the 3D architecture of the pelvic floor muscles in males and females, and to assess how normal variation in pelvic floor shape is associated with other biometric characteristics using a geometric morphometric approach. To this end, we conducted a comprehensive analysis of pelvic floor muscle structures. The shape of several pelvic floor muscles was quantified by a dense set of landmarks (3D measurement points) on CT scans of the pelvis in a cohort of deceased men and women. We then studied the association between biometric variables (parity, dimensions of the true pelvis, age, body weight and height) and pelvic floor muscle morphology in the two sexes.

Even though injuries and tissue weakness are the best recognized causes of PFD, we hypothesise that shape and size of the pelvic canal as well as overall muscle development contribute to variation in the shape and thickness of the pelvic floor muscles, which in turn affects PFD risk. Additionally, we expect that age, body weight and parity increase PFD risk mainly via their effect on pelvic floor morphology.

## Materials and methods

Computed tomography images from the New Mexico Decedent Image Database (‘NMDID’, https://nmdid.unm.edu/) of 49 males and 52 females between 20 and 60 years of age at death were used for this study. Exclusion criteria included signs of body decomposition, malignant disease, significant skeletal injuries or trauma to the pelvic area, and a BMI above 35. Average time between death and the CT scanning was 20.5 h. Landmarks were placed on CT images of the torso with a resolution of 0.5 x 0.5 x 0.5 mm, which is significantly higher than usual medical CT images available from live patients.

We focused on three groups of muscles: the levator ani, obturator internus and ischiocavernosus muscles. The levator ani layer covers the pelvic cavity from the front portion at the pubic bones to the rear, inserting into the coccyx, ischial spines and tendinous arches laterally. It is frequently subdivided into four parts: the puborectalis, a U-shaped sling that starts at the posterior pubis and inserts at the back of the rectum; the pubococcygeus, whose fibres start from the pubic bone and tendinous arch laterally to the puborectalis and insert into a midline raphe behind the rectum posteriorly; the iliococcygeus muscles, which arise from the lateral walls of the pelvis, run over the obturator internus to attach to the arcus tendineus, and insert into a midline raphe behind the rectum; and the coccygeus muscles, which attach anteriorly to the ischial spines and fan out medially to attach to the lateral surface of the coccyx [12]. The obturator internus muscles create an internal cushion of the anterior pelvic cavity overlaying the obturator foramen of the pelvis. The ischiocavernosus muscles belong to a more superficial layer of the pelvic floor and run parallel to the ischial rami. Together with the bulbocavernosus muscles, they are instrumental in sexual function and in supporting erection in both sexes as well as ejaculation in males.

**Fig. 1:**
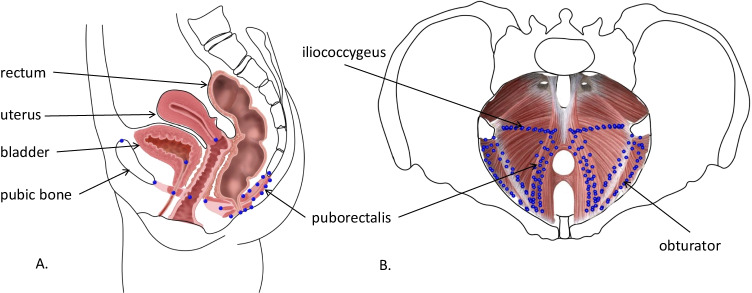
Location of landmarks, female configuration

**Fig. 2:**
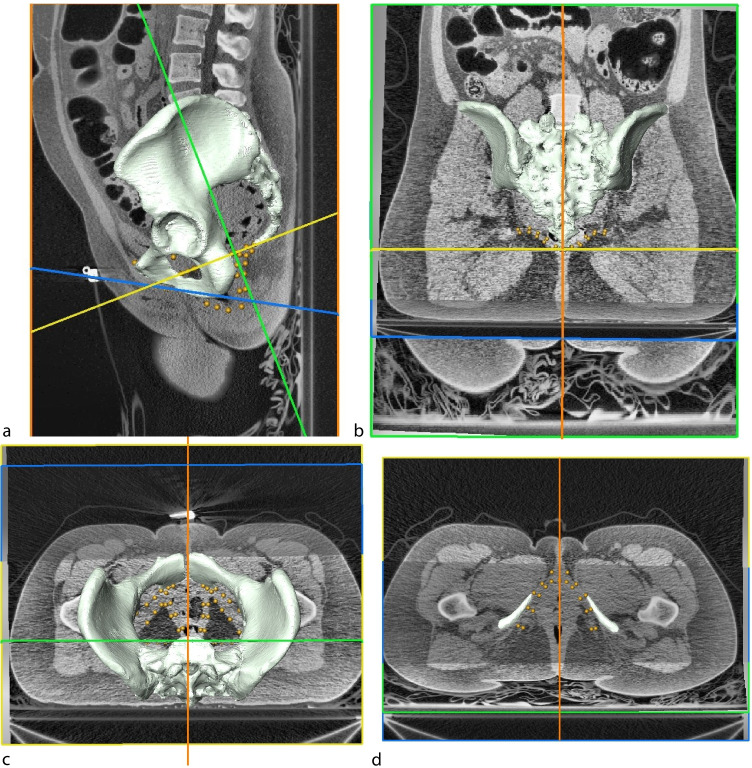
Slices used to place landmarks on outlines of pelvic floor muscles. The figure displays a 3D surface reconstruction of the pelvis from a CT scan and four slices through the CT stack used for collecting landmarks. *Orange* sagittal, *yellow* pubococcygeal, *green* iliococcygeal and *blue* ischiocavernosus slices. **a** Sagittal view; **b** posterior view; **c** superior view; **d** inferior view

We measured the pelvis and pelvic floor muscle outlines by collecting 3D landmarks and semilandmarks (196 in females and 194 in males, Fig. 1, Table S1) from the CT images [13, 14]. Qualitatively, male and female anatomy of the levator ani, obturator internus and ischiocavernosus are almost identical, despite differences in other structures. The same set of measured landmark points was therefore used in both sexes except for two extra points marking the position of the cervix and the vagina in females. The muscle outlines were measured by placing landmarks on oblique slices through the CT stack (Fig. 2). They corresponded in part to the standard urogynaecological planes [5, 15]:

1. Sagittal slice through the coccyx and pubis
2. Pubococcygeal slice, a plane perpendicular to the sagittal slice and passing through the lower pubic symphysis and the top of the ultimate coccyx vertebra
3. Iliococcygeal slice, a plane perpendicular to both the sagittal and to the pubococcygeal slices, and passing through the ischial spines
4. Ischiocavernosus slice, a plane passing through the widest cross-sections of the left and right ischiocavernosus muscles and aligned with the axes of the left and right ischial bones

After the landmark collection, we doubled the number of semilandmarks along the curves of interest to better capture the shapes of these curves. For that purpose, we fitted cubic splines to the original measurement points and then resampled points equidistantly along the cubic splines. The resulting set of curve-semilandmarks were slid along these curves by applying the sliding landmarks algorithm [16]. To standardise the landmark configurations to the same position, orientation and size, the data were subjected to generalised Procrustes analysis [13]. To reduce random noise in the data, we symmetrised landmark configurations by averaging each individual landmark configuration with its mirrored copy (relabelled reflection) [17]. The size of each individual was then restored by multiplying the shape coordinates with the centroid size of the respective individual. This produced form coordinates, which we used in the next step of data preparation. Centroid size is a standard measure of size in geometric morphometrics, computed as the square root of the sum of squared distances of all the landmark points of an individual from their centroid (mean of all landmark coordinates) [13].

In addition, we corrected for different degrees of rectum fill. The form coordinates were regressed on rectum size (measured as the geometric mean of the anteroposterior and mediolateral diameters of the rectum outline on the pubococcygeal slice). The residuals of this regression captured morphological variation in the landmark data independent of rectum fill. They were added to the mean landmark configuration and used in the next step of the analysis.

We calculated the anteroposterior (AP) depth of the pelvic outlet as the distance between the landmarks on the lower pubis and the fifth sacral vertebra. The mediolateral (ML) width of the pelvic midplane was obtained as the distance between the left and right ischial spines. Finally, muscle thicknesses were calculated as distances between the corresponding landmarks on the opposite outlines of each muscle (Table 1).

**Table 1:** Description of muscle thickness measurements

Muscle	Description
Obturator	Thickness at the middle of the muscle outline on the pubococcygeal slice, averaged across the left and right sides
Puborectalis	Thickness at the level anterior to the rectum on the pubococcygeal slice, averaged across the left and right sides
Sagittal puborectalis	Thickness of the posterior wall of the puborectalis above the anal sphincters on the sagittal slice
Iliococcygeus	Thickness at the middle of the muscle outline on the iliococcygeal slice, averaged across the left and right sides
Ischiocavernosus	Thickness at the middle of the muscle outline on the ischiocavernosus slice, averaged across the left and right sides

As independent variables, we included the age at death, body weight and body height at death as well as the number of live births for women, all obtained from the NMDID metadata. Additionally, we calculated the size of the lower pelvic canal, *R*, as the geometric mean of the ML and AP radii, and the shape of the canal as the AP/ML ratio. The overall muscular development of each individual was proxied by an independent measurement of the cross-sectional area of the thigh muscles (referred to as “muscular development” hereafter). For this we measured the thigh muscle area on a perpendicular cross-section of the thigh at the top of the linea aspera in the femur from the original CT scans. To be able to compare effect sizes for the different measures in the subsequent analyses, all independent variables were *z*-transformed.

We assessed the statistical significance of the overall multivariate association between the independent variables and the pelvic floor muscle shape coordinates by a permutation test, using the total shape variance explained by the independent variables as the test statistic and 5,000 random permutations. We then analysed the pattern of the association between the standardised shape landmark data (i.e. the data after the removal of noise due to the bilateral asymmetry and the rectum fill, and after a new superimposition with the help of generalised Procrustes analysis) and the independent variables using reduced rank regression (RRR). The latter is an exploratory method of decomposing the multivariate dependence of one set of variables on another set into some pairs (or “dimensions”) of linear combinations (latent variables) with maximal regression slopes [18]. In contrast to the more familiar partial least squares analysis (which maximises the covariance between latent variables), RRR maximises the regression slope, i.e. the effect of one unit change of the independent latent variable on the dependent latent variable. The corresponding loadings of the measured independent variables are partial regression coefficients, i.e. they quantify the effect of each measured variable on the landmark data, independent of the other variables. Technically, this is achieved by a singular value decomposition of the matrix of regression coefficients resulting from the multivariate multiple regression of the shape variables on the independent variables. In addition to regression analyses, a principal component analysis was run on the standardised shape landmark data to assess overall trends in morphology.

The association between muscle thicknesses and the independent variables was analysed for each muscle by multiple regression. As in the previous analyses, males and females were analysed separately. All calculations were performed in the statistical programming language R [19].

The current study did not require Ethics/Institutional Review Board (IRB) approval as it did not include live patients or animals.

## Results

### Pelvic floor muscle shape in males

The highest pairwise correlation among the independent variables was found between body weight and height (*r*=0.74, *p*<0.01, Table S2). The size of the lower pelvis, *R*, correlated moderately with the AP/ML ratio (*r*=0.44, *p*<0.01). Muscular development was positively associated with body weight and height (*r*=0.33, *p*=0.02 and *r*=0.44, *p*<0.01). Body weight increased with age (*r*=0.45, *p*<0.01). Figure S1 shows the distributions of all independent variables. The main axes of pelvic floor shape variance are depicted by the principal components in Figure S2. Neither age nor ethnicity explained the variation in pelvic floor muscle shape as shown by the first two principal components (Fig. S2).

**Fig. 3:**
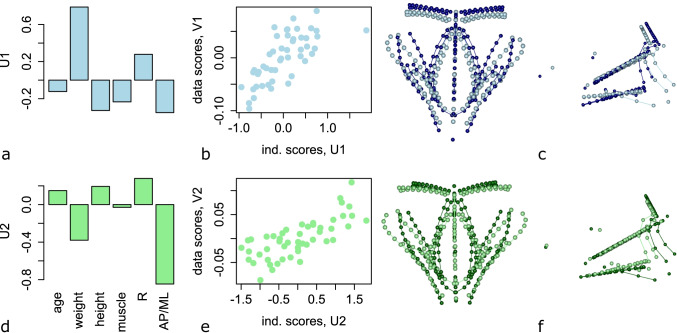
Results of the reduced rank regression (RRR) for males. RRR relates pelvic floor shape (as dependent variables) to the independent variables age, body weight, height, cross-sectional area of thigh muscles, pelvic canal size (*R*) and pelvic canal shape (anteroposteri﻿or [AP]/mediolateral [ML]; (see Materials and methods for details). This multivariate relationship is decomposed into two pairs of latent variables (rows 1 and 2). The strength of association is shown by the scatter plot of individual scores along dependent and independent patterns (**b**, **e**). The corresponding loadings for the independent variables (U1–2) are displayed as bar charts (**a**, **d**), and the loadings for pelvic floor shape (V1–V2) as actual shape deformations (**c**, **f**, superior and lateral views). The dark and light wireframes correspond to the smallest and largest individual scores of the corresponding shape pattern

A multivariate multiple regression of pelvic floor muscle shape on the independent variables explained 28% of the total shape variation in the sample. The permutation test of total shape variance explained by the independent variables was significant at *p*<0.001. The first pair of RRR vectors, U1 and V1, accounted for 58% of the regression effects (i.e. fraction of the first squared singular value and the sum of all squared singular values; Fig. S3). The vector U1 contains loadings (partial regression coefficients) of the independent variables on the first latent variable, and V1 comprises the loadings of the landmark shape coordinates, which can be visualised as an actual pelvic floor shape deformation. For our data, the first RRR dimension was dominated by a high positive loading of body weight (Fig. 3a). The corresponding shape loadings are visualised in Fig. 3c: high body weight in males is associated with an anteriorly shifted iliococcygeus muscle, thinner obturator muscles, and a deeper pelvic floor due to a considerably longer rectum.

The second pair of RRR vectors accounted for 19% of the summed squared regression effects (Fig. S3) and was dominated by a high loading of pelvic canal shape (AP/ML). The outline of the pelvic floor muscles followed the shape of the lower pelvis closely, with a wider pelvis associated with a relatively shorter rectum (Fig. 3d−f).

### Pelvic floor muscle shape in females

As in males, the highest correlation in females was found between body weight and height (*r*=0.54, *p*<0.01, Table S3) as well as between body weight and muscular development (*r*=0.58, *p*<0.01). Age had a moderate negative correlation with both height and muscular development (*r*=−0.36, *p*=0.01, and *r*=−0.32, *p*=0.02). The shape of the lower pelvis (AP/ML) showed a positive correlation with body weight (*r*=0.41, *p*<0.01), where the pelvis was anteroposteriorly longer in heavier individuals (Table S3). Figure S4 shows the distribution of the independent variables. The main axes of pelvic floor shape variation are depicted in Figure S5. Unlike in males, a clear association between age and the first two principal components of pelvic floor muscle shape variation is visible. Young females tend to cluster at higher scores for PC1 and at lower scores for PC2, compared with older females.

**Fig. 4:**
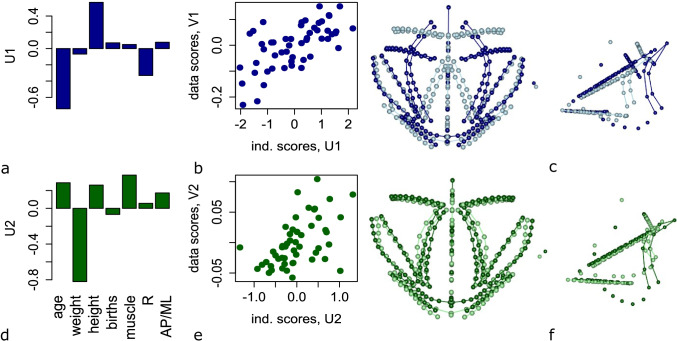
Results of the reduced rank regression (RRR) for females. RRR relates pelvic floor shape (as dependent variables) to the independent variables age, body weight, height, cross-sectional area of thigh muscles, pelvic canal size (*R*) and pelvic canal shape (anteroposterior [AP]/mediolateral [ML]). This multivariate relationship is decomposed into two pairs of latent variables (rows 1 and 2). The strength of association is shown by the scatter plot of individual scores along dependent and independent patterns (**b**, **e**). The corresponding loadings for the independent variables (U1–2) are displayed as bar charts (**a**, **d**), and the loadings for pelvic floor shape (V1–V2) as actual shape deformations (**c**, **f**, superior and lateral views). The dark and light wireframes correspond to the smallest and largest individual scores of the corresponding shape pattern

The multivariate multiple regression accounted for 28% of the total variance in female pelvic floor shape (*p*=0.001). The first pair of RRR vectors accounted for 42% of the summed squared regression coefficients (Fig. S6). Age and pelvic canal size showed high negative loadings on the first RRR dimensions, whereas body height had a positive loading. As these are partial regression coefficients, a positive loading for height independent of body weight implies a low body mass index (weight relative to height). Advanced age, a large pelvic canal and low body height relative to weight were associated with a considerably wider puborectalis sling, a downwardly “sagged” iliococcygeus muscle and a thinner posterior wall of the puborectalis (Fig. 4a−c).

The second pair of RRR vectors accounted for 24% of summed squared regression effects (Fig. S6). It showed that women with high relative body weight tend to have a deeper pelvic floor due to a relatively longer rectum and a lower position of the perineal area (Fig. 4d−f, dark green colour).

### Muscle thickness

**Fig. 5:**
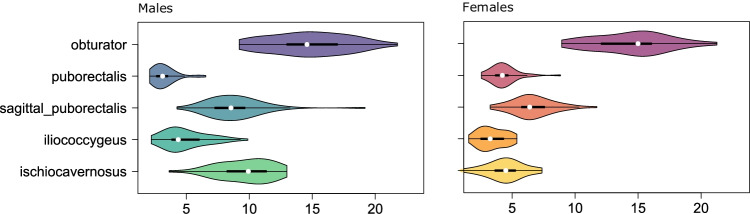
Distributions of muscle thicknesses in males and females. The *white dots* are medians, and the central 50% of the distributions are indicated by the *thick black lines*. *Thin black lines* depict the 25th and 75th percentiles

Most pelvic floor muscle were thicker in males than in females. The puborectalis muscles, however, were thicker in females when measured on the pubococcygeal slice anterior to the rectum. The largest difference between men and women was found in the thickness of the ischiocavernosus muscles, which were on average almost twice as thick in men. The obturator muscles of the two sexes were similar (Table 2, Fig. 5).

**Table 2:** Muscle thicknesses in males and females

	Males				Females			
	Minimum (mm)	Maximum (mm)	Mean (mm)	SD	Minimum (mm)	Maximum (mm)	Mean (mm)	SD
Obturator	9.2	21.8	14.7	3.07	9.0	21.3	14.5	2.92
Puborectalis	2.1	6.5	3.2	0.95	2.6	8.8	4.3	1.09
Sagittal puborectalis	6.0	19.6	9.5	2.30	3.3	11.7	6.6	1.66
Iliococcygeus	2.2	9.9	4.9	1.73	1.6	5.4	3.4	1.05
Ischiocavernosus	3.6	13.0	9.6	2.12	0.9	7.4	4.4	1.47

In males, the multiple regression of obturator muscle thickness on the independent variables explained 25% of its variation in our sample (see Table S4) and was dominated by the effects of body weight and muscular development. In addition, muscle development was positively associated with the thickness of the iliococcygeus muscle (Table S4). Age affected the thicknesses of the iliococcygeus and ischiocavernosus muscles in men. However, the direction of these effects differed: the iliococcygeus became slightly thicker with age, although the ischiocavernosus thickness decreased with age (Table S4).

In females, the obturator muscle thickness was positively associated with the size of the lower pelvic canal (Table S5). Women with a larger number of children also tended to have thicker obturator muscles, as well as thicker puborectalis muscles (Table S5). Unlike in men, sagittal thickness of the puborectalis muscle in women significantly decreased with age. The iliococcygeus muscle, on the contrary, slightly increased in thickness with age in women, whereas ischiocavernosus muscle thickness was not affected by age. Finally, the size of the lower pelvic canal had a similar positive effect on the thickness of the puborectalis muscle on the pubococcygeal slice in men and in women (Table S4 and S5).

## Discussion

Age had the strongest effect on the shape of the female pelvic floor muscles. Older women, on average, had a wider distance between the left and right parts of the puborectalis muscle (i.e. a wider genital hiatus), a more caudal orientation of the iliococcygeus muscles and a thinner posterior wall of the puborectalis muscle (Figs. 3, S7). However, age did not affect the positions of the cervix and bladder neck, in correspondence with previous findings by Wu et al. [20]. Body height and body weight also had strong effects on the shape of the pelvic floor muscles in women. Women with a higher weight relative to body height had a longer rectum and a considerably lower position of the perineal area, cervix and bladder neck compared with lighter women (Fig. 3). Surprisingly, parity did not have a strong association with the overall shape of the pelvic floor muscles.

The significant impact of age on pelvic floor muscle structure and function has been reported by a previous study [21]. Age-related changes were suggested to be a consequence of alterations in the structure of skeletal muscles and a progressive denervation of the pelvic floor. Swenson et al. [21] found that in nulliparous women, age affects the shape of the genital hiatus, which is created by the puborectalis muscles wrapped around the pelvic floor organs: younger women show a V-shape, whereas older women tend to have a wider, more open U-shape of the hiatus.

In males, body weight had the strongest effect on the shape of the pelvic floor muscles. Large body weight was associated with a mediolaterally wider pelvis that supported a longer rectum and therefore a longer perineal area (Fig. 3a−c). Compared with females, age had a small effect on pelvic floor muscle shape in males (Fig. 3a−f) but was strongly associated with reduced thickness of the ischiocavernosus muscles (Table S4).

Men had thicker pelvic floor muscles, except for the puborectalis muscles, which were thicker in women. This sex difference could be due to an evolutionary adaptation for childbirth in females (Table 2, Fig. 4). The largest difference between the sexes was found in the ischiocavernosus muscles, in agreement with the higher functional load on this muscle group in males.

Our finding that parity does not affect the global 3D shape of the female pelvic floor muscles seems surprising at first glance, given that parity and vaginal delivery are the main risk factors of pelvic organ prolapse [2, 3]. However, the main causes of birth-related PFDs are injury and avulsion of the puborectalis and pubococcygeal muscles, which may simply not be visible on the CT scans. Birth-induced injuries mostly occur at the pubovisceral portion of the levator ani and are detectable on MRI in only 55% of women with prolapse, whereas the puborectal part–the part that we measured in this study–does not show injuries in cases of pelvic organ prolapse [22]. The lack of association between parity and pelvic floor muscle shape in this study may thus imply that local birth injuries, despite their importance for pelvic floor health, do not affect the global geometry of the pelvic floor muscles.

Sammarco et al. [23] demonstrated that women with pelvic floor prolapse frequently have a larger anterior pelvic floor area and wider interspinous diameters. Berger et al. [24] found that young primiparous women with urinary stress incontinence tend to have wider interspinous diameters than continent women. Similar associations between a large lower pelvic canal and increased risk of PFDs were reported by other studies [7]. Also, previous biomechanical modelling showed that a large pelvic floor provides weaker support and descends deeper on application of intra-abdominal pressure than does a smaller one [9]. In agreement with these clinical and biomechanical results, we found that a relatively larger lower pelvic canal in females was associated with a deeper pelvic floor and a wider puborectalis sling (Fig. 4a, b). Such a descent may be counteracted by an increased thickness of the pelvic floor muscles [9]. In the present study, we found that the size of the lower pelvic canal (*R*) correlates positively with the thickness of the puborectalis muscle in both men and women (Tables S4 and S5), suggesting that the disadvantages of the larger pelvic floor might indeed be ameliorated by thicker pelvic floor muscles. The association between pelvic canal size and the risk of PFDs has been intensively discussed in evolutionary anthropology because such an association imposes natural selection toward a narrow birth canal and explains, at least partly, why the human birth canal did not evolve to be wider [25].

In this study, we assessed for the first time the association of the 3D morphology of pelvic floor muscles with several biometric variables in men and women. Most clinical investigations concentrated on 2D measurements from MRI, for example, the position of the internal organs above the pubococcygeal axis, the muscle thickness on transverse, coronal or sagittal views of MRI [26], thicknesses of adjacent musculature by ultrasound [27] or thickness and micro-morphology of prepared cadaver tissue [28]. We proposed a novel approach for a comprehensive quantification of the 3D morphology of the pelvic floor muscles in males and females, which may also be applicable in clinical practice.

Capturing pelvic floor muscle shape is limited by the malleable quality of the soft tissue. For example, muscular contraction and the degree to which the rectum and bladder are filled alter the shape of the pelvic floor. Similarly, body position and intra-abdominal pressure affect the position of the internal organs and consequently pelvic floor muscle shape [29]. We partly addressed these issues by statistically controlling for variation in rectum diameter due to its content. The clinical standard of quantifying pelvic organ descent is the examination of a woman in the dorsal lithotomy position, prompting her to Valsalva, and then measuring organ descent, according to the Pelvic Organ Prolapse Quantification Score [30]. By contrast, our data were collected from CT scans of deceased persons in prone position, which constitutes a clear limitation of this study. However, both the preservation of the deceased bodies and the scanning position were standardised in our sample, implying that individual differences in pelvic floor muscle morphology between individuals could be meaningfully measured and analysed. In addition, because of the standardised preservation of the deceased bodies, differences in intra-abdominal pressure and muscle tone were negligible. We suggest that future studies should be conducted on living patients to confirm the findings of this study.

## Conclusions

We found that age, body weight relative to body height, and the size of the lower pelvic canal affect the shape of the pelvic floor muscles. High body weight leads to a more caudal position of the pelvic floor in both sexes, whereas the effect of age is much stronger in women than in men. In men, age affects the thickness of the ischiocavernosus muscles, which are important for sexual function. Contrary to our expectations, parity was not clearly associated with global pelvic floor shape in women. We therefore suggest that the risk of PFDs resulting from vaginal delivery originates primarily from local pelvic floor injury and muscle avulsion rather than from a change in the global geometry of the pelvic floor. Independently of childbirth, the pelvic floor tends to become oriented more caudally with increasing age in women, which may indicate muscle atrophy that increases the risk of PFDs.

## Supplementary information

ESM 1 (PDF 785 kb)
