# Supplementary material for: The variation in shape and thickness of the pelvic floor musculature in males and females: a geometric-morphometric analysis
Source: Int Urogynecol J. 2022 Aug 5;34(2):453–61. doi: 10.1007/s00192-022-05311-5 (PMC9870833; doi:10.1007/s00192-022-05311-5)

Supplementary Material

Table S1

name	*description*	*male CT*	*femal e CT*
	1. SAGITTAL SLICE		
Upper Pubic Symphysis		X	X
Lower Pubic Symphysis		X	X
Coccyx		X	X
Urethra	centre of the urethra below the pubic symphysis	X	X
Vagina	centre point of the shadow marking vagina		X
Anus	centre point of the anal sphincter	X	X
Pubococcygeus anterior	5 lms – from coccyx to anus	X	X
Pubococcygeus posterior	5 lms – from coccyx to anus	X	X
Perineal body	3 lms -- from anus to urethra in males and anus to vagina in females	X	X
Cervix			X
Bladder neck		X	X
	2. PUBOCOCCYGEUS SLICE		
Left obturator lateral	5 lms -- from the posterior part of pubis to ischium up to an obvious insertion point	X	X
Left obturator medial	5 lms -- perpendicular to the first point to reflect thickness, up to the corner with the fatty tissue. If there is no clear corner, finish at the level with the end of ischium	X	X
Right obturator lateral	5 lms -- from the posterior part of pubis to ischium up to an obvious insertion point	X	X
Right obturator medial	5 lms -- perpendicular to the first point to reflect thickness, up to the corner with the fatty tissue. If there is no clear corner, finish at the level with the end of ischium	X	X
Left puborectalis lateral	5 lms – start from the pubic bone, 5 lms, finish before reaching central line.	X	X
Left puborectalis medial	5 lms – start from the pubic bone, follow the brightest path, finish before the central line.	X	X
Right puborectalis lateral	5 lms – start from the pubic bone, 5 lms, finish before reaching central line.	X	X
Right puborectalis medial	5 lms – start from the pubic bone, follow the brightest path, finish before the central line.	X	X

	3. ILIOCOCCYGEUS SLICE		
Left iliococccygeal top	5 lms– start as close to the bone as the muscle is clearly separated from connective tissue, finish before reaching coccyx	X	X
Left iliococccygeal bottom	5 lms – start as close to the bone as the muscle is clearly separated from connective tissue, finish before reaching coccyx	X	X
Right iliococccygeal top	5 lms– start as close to the bone as the muscle is clearly separated from connective tissue, finish before reaching coccyx	X	X
Right iliococccygeal bottom	5 lms – start as close to the bone as the muscle is clearly separated from connective tissue, finish before reaching coccyx	X	X
	4. ISCHIOCAVERNOSUS SLICE		
Central point	at the penis/clitoris base		
Left ischiocavernosus lateral	5 lms. The first lm is to be placed at the base of the penis/clitoris on the outside. It is usually higher than the central point. The line central point-first landmark should be approximately perpendicular to the tangent to the curve here.	X	X
Left ischiocavernosus medial	5 lms. The first lm is placed between the central point and the level of the ischial bone. The second landmark should be approximately across the second landmark of the outside curve	X	X
Right ischiocavernosus lateral	5 lms. As above	X	X
Right ischiocavernosus medial	5 lms. As above	X	X

Table S2. Correlation coefficients for independent variables. Males. Significance: (**) at 0.01 level, (*) at 0.05 level, (.) at 0.1 level.

p-val. / *Correl.*	*Age*	*weight*	*height*	*muscle*	*R*	*A/ML*
age		**<0.01****	**0.09.**	0.35	0.32	0.79
weight	**0.45**		**<0.01****	**0.02***	0.80	0.11
height	**0.24**	**0.74**		**<0.01****	0.57	0.91
muscle	-0.14	**0.33**	**0.44**		0.19	0.53
R	-0.14	-0.04	-0.08	-0.19		**<0.01**
AP/ML	0.04	0.23	0.02	-0.09	**0.44**	

Fig S1. Histograms for distributions of independent variables in males.

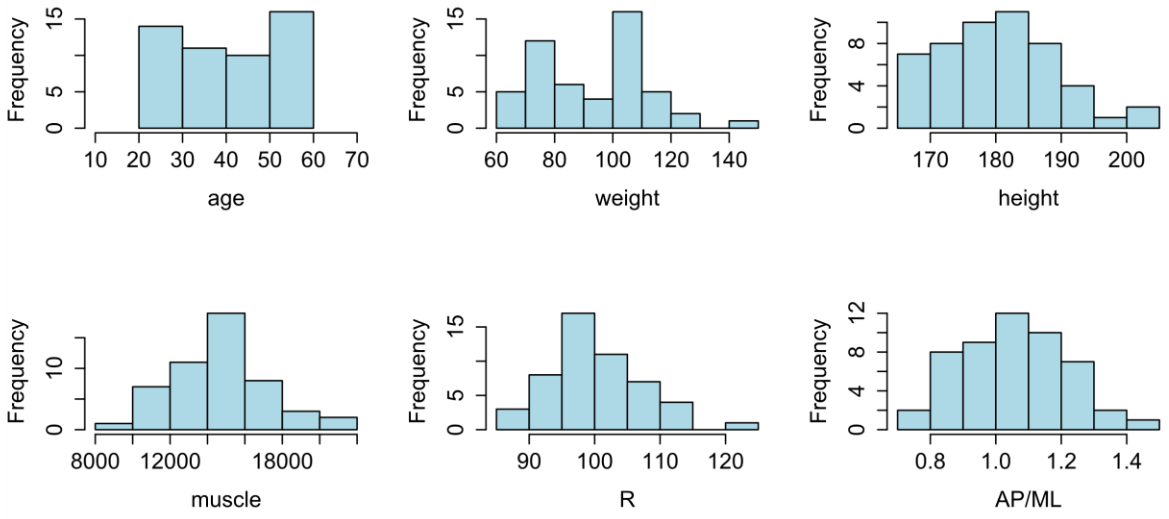

Fig S2. Principal component analysis of pelvic floor shape in males. Colours correspond to age: from dark blue at 20 to yellow at 60 years of age. Wireframes for PC1 and PC2 are colour coded: light colour corresponds to the maximum positive value; dark colour corresponds to the minimum negative value.

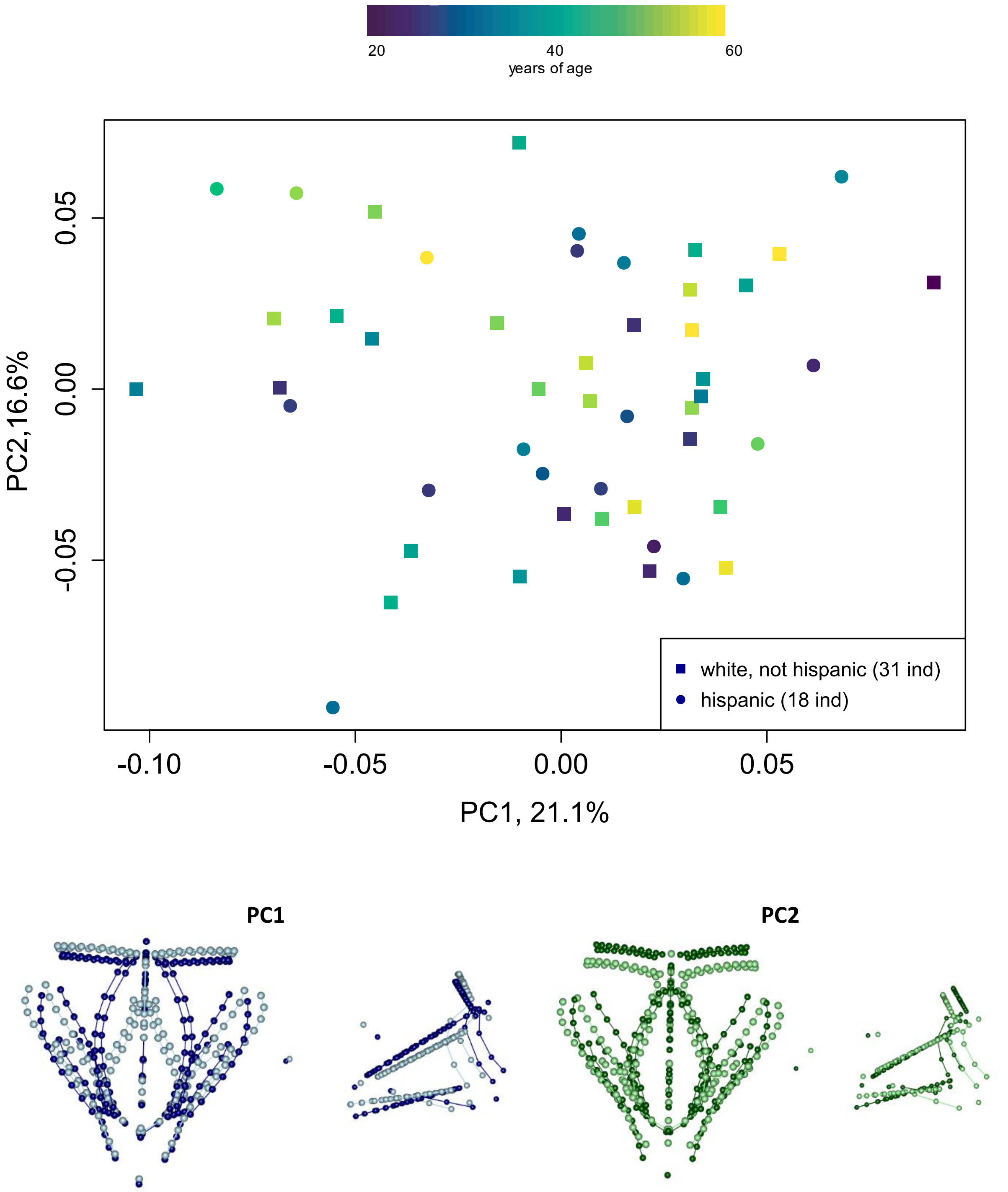

Figure S3. Share of total regression effect, in %, reduced rank regression vector pairs for males.

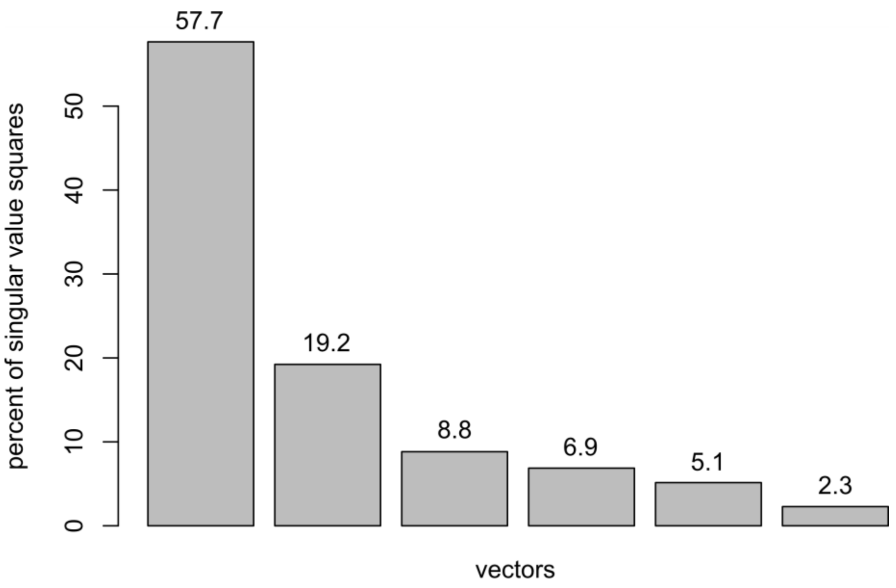

Table S3. Correlation coefficients for independent variables. Females. Significance: (**) at 0.01 level, (*) at 0.05 level, (.) at 0.1 level.

p-val. / *correl.*	*age*	*weight*	*height*	*births*	*muscle*	*R*	*AP/ML*
age		0.36	**0.01****	0.12	**0.02***	0.64	0.72
weight	-0.13		**<0.01****	**0.04***	**<0.01****	**0.02***	**<0.01****
height	**-0.36**	**0.54**		**0.01****	0.17	0.18	**0.05***
births	0.22	**-0.28**	**-0.38**		0.31	0.66	0.68
muscle	-0.32	**0.58**	0.19	-0.14		0.43	0.28
R	0.07	**0.33**	0.19	-0.06	0.11		0.77
AP/ML	0.05	**0.41**	**0.28**	-0.06	0.15	-0.04	

Fig S4. Histograms for distributions of independent variables in females.

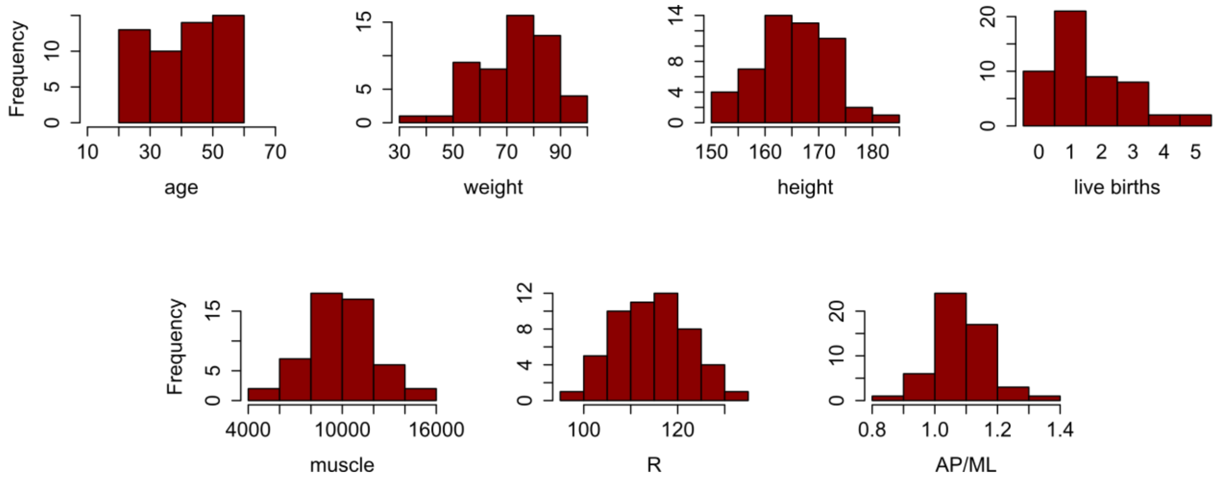

Fig S5. Principal component analysis of pelvic floor shape in females. Colours correspond to age: from dark blue at 20 to yellow at 60 years of age. Wireframes for PC1 and PC2 are colour coded: light colour corresponds to maximum positive value; dark colour corresponds to minimum negative value.

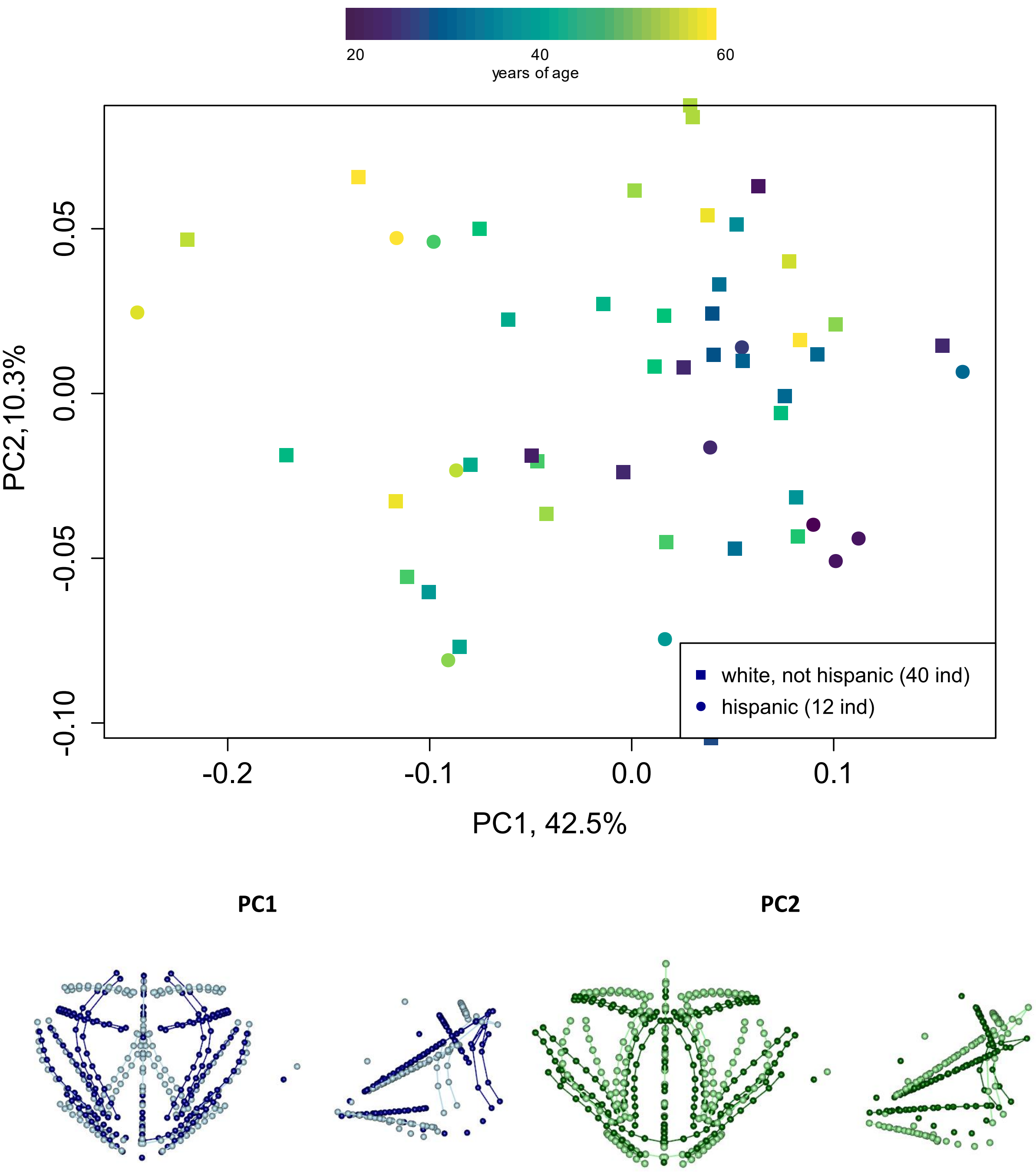

Figure S6. Share of total regression effect, in %, in the reduced rank regression for females.

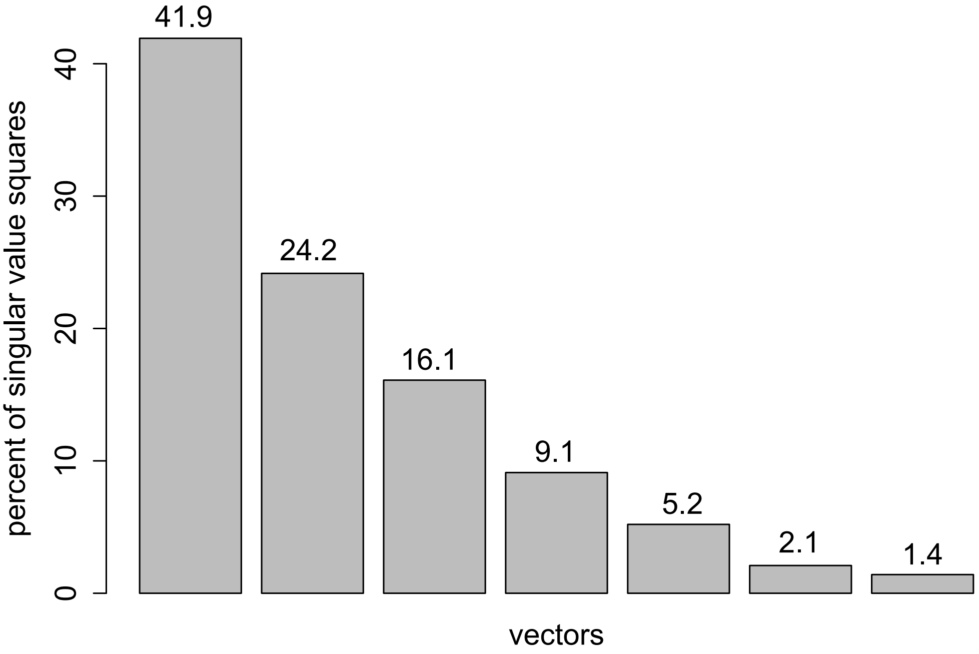

Table S4. Males. Results of regression of pelvic floor muscle thicknesses on each of the independent variables separately. Significance: (**) at 0.01 level, (*) at 0.05 level, (.) at 0.1 level.

**obturator**						
	**age**	**weight**	**height**	**muscle**	**R**	**AP/ML**
coefficient	0.11	**-1.68**	0.25	**1.13**	-0.52	0.52
p-value	0.81	**0.02**	0.69	**0.02**	0.28	0.30
		*		*		
Multiple R^2:		0.252		Multiple p-value:		0.038
**sagittal_puborectalis**						
	**age**	**weight**	**height**	**muscle**	**R**	**AP/ML**
coefficient	-0.10	0.81	-0.52	**0.79**	-0.14	-0.09
p-value	0.79	0.16	0.31	**0.05**	0.71	0.81
				*		
Multiple R^2:		0.194		Multiple p-value:		0.148
**puborectalis**						
	**age**	**weight**	**height**	**muscle**	**R**	**AP/ML**
coefficient	0.25	0.23	-0.12	0.04	**0.30**	0.26
p-value	0.16	0.39	0.59	0.81	**0.08**	0.17
					.	
Multiple R^2:		0.291		Multiple p-value:		0.015

iliococcygeus

	age	weight	height	muscle	R	AP/ML
coefficient	**0.49**	-0.18	-0.11	**0.52**	-0.42	0.19
p-value	**0.08**	0.66	0.76	**0.05**	0.12	0.50
	.			*		
Multiple R^2:		0.197		Multiple *p*-value:		0.122

ischiocavernosus

	age	weight	height	muscle	R	AP/ML
coefficient	**-0.86**	-0.06	-0.17	0.14	-0.04	-0.20
p-value	**0.01**	0.90	0.70	0.65	0.91	0.57
	**					
Multiple R^2:		0.225		Multiple *p*-value:		0.069

Table S5. Females. Results of regression of pelvic floor muscle thicknesses on each of the independent variables separately. Significance: (**) at <0.01 level, (*) at 0.05 level, (.) at 0.1 level.

obturator

	age	weight	height	births	muscle	R	AP/ML
coefficient	0.02	0.67	0.08	**0.75**	0.21	**1.18**	0.05
p-value	0.97	0.27	0.88	**0.06**	0.67	**<0.01**	0.91
				.		**	
Multiple R^2:		0.331		Multiple *p*-value:			0.009

sagittal puborectalis

	age	weight	height	births	muscle	R	AP/ML
coefficient	**-0.58**	0.06	-0.29	0.11	-0.12	-0.05	0.18
p-value	**0.05**	0.88	0.37	0.67	0.72	0.85	0.52
	*						
Multiple R^2::		0.102		Multiple *p*-value:			0.658

puborectalis

	age	weight	height	births	muscle	R	AP/ML
coefficient	-0.17	-0.17	0.13	**0.35**	-0.04	0.24	0.17
p-value	0.34	0.49	0.52	**0.04**	0.83	0.15	0.35
				*			
Multiple R^2::		0.161		Multiple *p*-value:			0.321

iliococcygeus

	**age**	**weight**	**height**	**births**	**muscle**	**R**	**AP/ML**
coefficient	**0.29**	0.12	0.17	0.24	0.20	-0.25	0.04
p-value	**0.10**	0.62	0.39	0.13	0.31	0.12	0.81
	.						
Multiple R^2::		0.185		Multiple *p*-value:			0.220

ischiocavernosus

	**age**	**weight**	**height**	**births**	**muscle**	**R**	**AP/ML**
coefficient	-0.07	0.32	-0.21	-0.07	0.03	-0.26	0.06
p-value	0.79	0.38	0.48	0.76	0.92	0.28	0.81
Multiple R^2::		0.065		Multiple *p*-value:			0.871

Figure S7. Comparison of the pelvic floor muscles shape in a young and an older woman. The shape of the puborectalis muscle is looser in the 58-year-old woman.

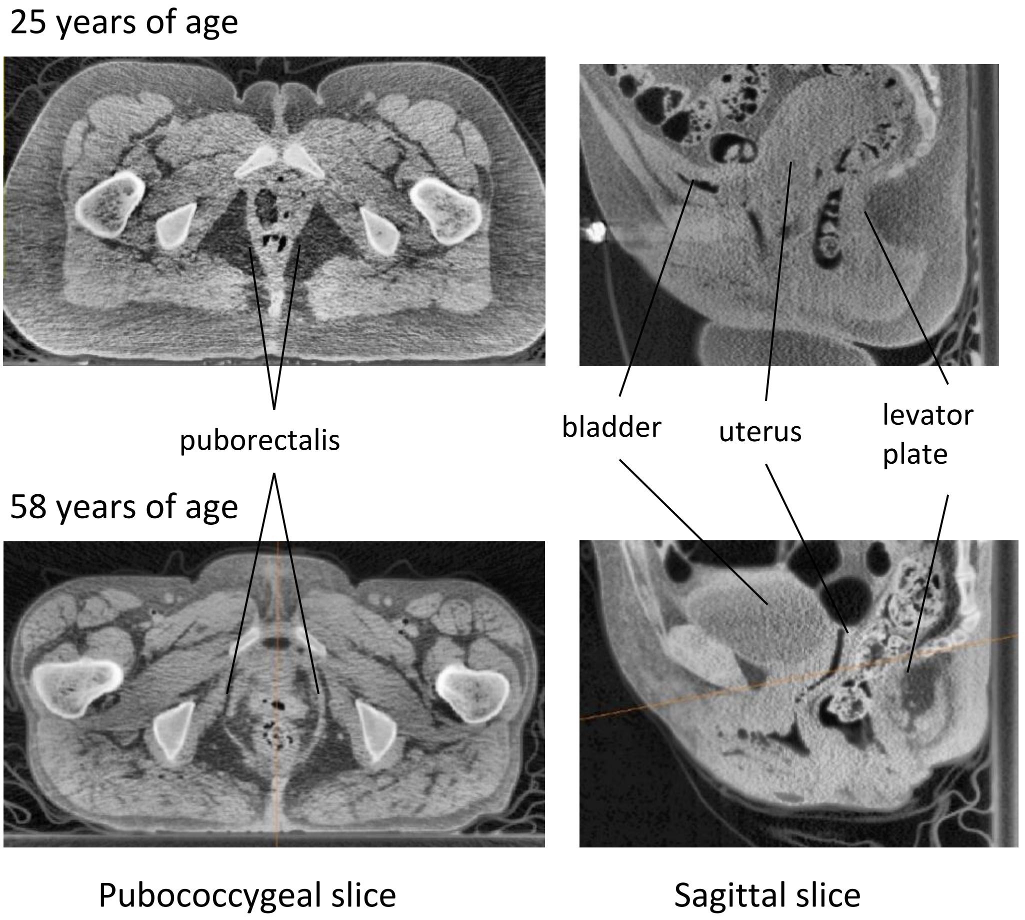

Supplement: Supplementary file 1 — (PDF 785 kb) [file 192_2022_5311_MOESM1_ESM.pdf]
